# The relationship between complement C1q and coronary plaque vulnerability based on optical coherence tomography analysis

**DOI:** 10.1038/s41598-024-60128-0

**Published:** 2024-04-25

**Authors:** Yuan Wang, Jiawei Zheng, Qing Li, Yao Ma, Chang Liu, Jie Deng, Dengfeng Gao

**Affiliations:** Department of Cardiology, The Second Affiliated Hospital, Xi’an Jiaotong University, No. 157, Xiwu Road, Xi’an, 710000 Shaanxi People’s Republic of China

**Keywords:** Coronary artery disease, Complement C1q, Optical coherence tomography, Biomarkers, Cardiology

## Abstract

To determine the association between complement C1q and vulnerable plaque morphology among coronary artery disease (CAD) patients. We conducted a retrospective observational study of 221 CAD patients admitted to The Second Affiliated Hospital of Xi’an Jiaotong University. Intravascular optical coherence tomography was utilized to describe the culprit plaques’ morphology. Using logistic regression analysis to explore the correlation between C1q and vulnerable plaques, and receiver operator characteristic (ROC) analysis assess the predictive accuracy. As reported, the complement C1q level was lower in ACS patients than CCS patients (18.25 ± 3.88 vs. 19.18 ± 4.25, *P* = 0.045). The low complement-C1q-level group was more prone to develop vulnerable plaques. In lipid-rich plaques, the complement C1q level was positively correlated with the thickness of fibrous cap (r = 0.480, *P* = 0.041). Univariate and multivariate logistic regression analyses suggested that complement C1q could be an independent contributor to plaques’ vulnerability. For plaque rupture, erosion, thrombus, and cholesterol crystals, the areas under the ROC curve of complement C1q level were 0.873, 0.816, 0.785, and 0.837, respectively (*P* < 0.05 for all). In CAD patients, the complement C1q could be a valuable indicator of plaque vulnerability.

## Introduction

Coronary artery disease (CAD) is a chronic, progressive disease that can be classified into acute coronary syndrome (ACS) and chronic coronary syndrome (CCS)^1^. Atherosclerosis is an inflammatory/immune disease that triggers the development of plaques at particular locations within the arterial tree^2^. Previous studies have demonstrated that the complement system is recognized as pro-inflammatory and that complement activation is associated with the progression of atherosclerosis and its complications^3^. However, as a crucial element of the complement system, C1q plays both protective and pathological roles in atherosclerosis^4^. Some reports have demonstrated that C1q could be an initiating factor of the classical pathway, which retards the formation of atherosclerotic plaques by promoting the removal of apoptotic cells in early atherosclerosis^5^. In addition, recent clinical studies have demonstrated that complement C1q, which may be a reflection of inflammation reaction grade during atherosclerosis, is an excellent predictor of risk for cardiovascular disease^6^.

Vulnerable atherosclerotic plaques are usually responsible for major adverse cardiovascular events^7^, which have distinctive features for identification on OCT, including large necrotic cores, thin fibrous caps, microcalcifications, intraplaque haemorrhage, neoangiogenesis, and inflammatory cell infiltration^8^. The predominant mechanism leading to acute coronary syndrome is thought to be the formation of occlusive thrombus on vulnerable plaques due to plaque rupture, plaque erosion, and calcified nodules^9^. Key to promoting the prevention of adverse cardiovascular events and improving therapy is the early recognition of vulnerable atherosclerotic plaques. Inflammation is the main mechanism participating in the formation, development, instability, and healing of atherosclerotic plaques^10^. Pathological research has also revealed a large infiltration of inflammatory cells at the site of plaque rupture^11^. These studies show that inflammation is strongly correlated with the vulnerability of plaques.

However, the correlation between the complement C1q level and plaque vulnerability has yet to be adequately elucidated. In this regard, the current study's objective was to clarify the detection value of the complement C1q level for plaque vulnerability measured by OCT in CAD patients and to explore whether the complement C1q level could be utilized as an indicator of coronary plaque instability to help individualize secondary prevention strategies.

## Methods

### Study population

In this study, 221 consecutive patients with CAD aged ≥ 18 years who underwent simultaneous coronary angiography and OCT evaluation between April 2021 and July 2022 at the Second Affiliated Hospital of Xi'an Jiaotong University were retrospectively recruited. Individuals or lesions were disqualified if at least one of the following conditions was true: (I) a history of coronary artery bypass surgery; (II) extreme tortuosity, left-main stenosis, severe calcification, or chronic complete occlusion for potential difficulty in performing OCT examination; (III) severe liver dysfunction or serious kidney disease (effect glomerular filtration rate/eGFR < 30 ml/ (min⋅1.73 m2); and (IV) autoimmune system diseases or long-term use of immunosuppressive drugs for other factors.

In accordance with ASCVD guidelines. ACS includes ST-segment elevation myocardial infarction (STEMI), non-ST-segment elevation myocardial infarction (NSTEMI) and unstable angina (UA). CCS refers to the different stages of ASCVD, such as (i) patients with ‘stable’ anginal symptoms; (ii) asymptomatic and symptomatic patients > 1 year after initial diagnosis or revascularization; (iii) patients with suspected vasospastic or microvascular disease, but excluding the situations of ACS^1^.

Clinical characteristics, including age, sex, BMI, history of previous medication (diagnosed as hypertension or diabetes), and current medication (including statins, aspirin, etc.), were extracted from the electronic medical record system. Body mass index (kg/m^2^) was derived by dividing weight in kilograms by height in metres squared (kg/m^2^). The study was conducted in accordance with the Declaration of Helsinki (as amended in 2013). The Second Affiliated Hospital of Xi'an Jiaotong University's institutional ethics committee authorized this research (registration number is 2,021,056). Confirms that informed consent was obtained from all participants.

### Blood collection

When patients were hospitalized, blood samples were collected and taken into tubes containing 0.1% EDTA for serum analyses at the first time. Serum complement C1q (mg/L) levels were measured by immunity transmission turbidimetry using Siemens BNII instrument, the concentrations of relevant lipoprotein markers, such as total cholesterol (TC) (mmol/L), total triglycerides (TG) (mmol/L), LDL-C (mmol/L), HDL-C (mmol/L), very-low-density lipoprotein cholesterol (VLDL-C) and Lipoprotein (a) (mg/dL) were measured by electro-chemiluminescence immunoassay (Roche Diagnostics, Indianapolis, IN). Plasma haemoglobin A1c (HbA1c) (%), creatine (CREA) (μmol/L) and other laboratorial parameters were measured by a biochemical analyzer according to standard test protocols (Hitachi-7600, Tokyo, Japan).

### OCT imaging and analysis

When diagnostic coronary angiography was performed, OCT imaging of culprit lesions was acquired by commercially accessible frequency-domain OCT equipment (Mobile Dragonfly, St. Jude Medical/Abbott, St. Paul, MN, USA). Once 0.2 mg of nitroglycerin was injected intracoronarily, the culprit lesion's distal end was carefully approached using a 2.7F OCT imaging catheter. After a brief injection of contrast medium through a guide catheter for blood flushing, the catheter was automatically withdrawn at a speed of 20 mm/sec. Thrombus aspiration and/or gentle dilatation with a tiny balloon was applied to acute entirely occluded lesions or severely stenosis lesions. Images are stored digitally, and OCT findings can be analysed using the self-contained system software after two or more experienced physicians who were blinded towards the angiographic statistics and clinical presentations have verified the quality of the images obtained.

OCT images were evaluated according to the expert consensus^12^, which measured the lesioned vessel and the different types of plaques. Two interventional cardiovascular pathologists independently analysed the OCT images, and the accuracy and consistency of the manual analysis were 98.9% and 99.3%, respectively. The performance of different culprit plaques on OCT images varied, the minimum lumen area and lumen diameter refer to the minimum area and diameter of the coronary lumen in the presence of a plaque, and the minimum lumen diameter and the minimum lumen area of the coronary vessel are recorded separately. Using the system measurement software, the thinnest thickness of the fibrous cap of the vulnerable plaque was measured 3 times, and the mean value was considered the thinnest fibrous cap thickness (FCT) of this plaque; then, the minimum lumen diameter, minimum lumen area, lipid pool curvature, lipid core length, and lipid index of this plaque were recorded.

### Statistical analysis

Data of continuous variables were expressed as the mean ± standard deviation (SD). Independent samples t tests or Mann‒Whitney tests were utilized to compare differences between two groups. Categorical variables were stated as counts and percentages (%) and compared using the chi-square test or Fisher's exact test. Spearman's correlation analysis was employed to identify the relationship between serum complement C1q values and the thickness of the fibrous cap on the lipid plaque. After adjusting for confounders, logistic regression analysis was applied to investigate the connection between complement C1q levels and vulnerable plaques. Receiver operating characteristic (ROC) curves were generated to analyse the correlation between plaque vulnerability characteristics and complement C1q levels, and the area under the curve (AUC) served as a measure of predictive accuracy. A *P* value of < 0.05 was considered to indicate statistical significance. All analyses were performed using Empower Stats software (R) (www.empowerstats.com, X&Y Solutions, Inc. Boston, MA), R software (version 3.2.0), and SPSS 26.0 software (SPSS Inc, Chicago, IL, USA).

## Results

### Characteristics of patients

**Figure 1:**
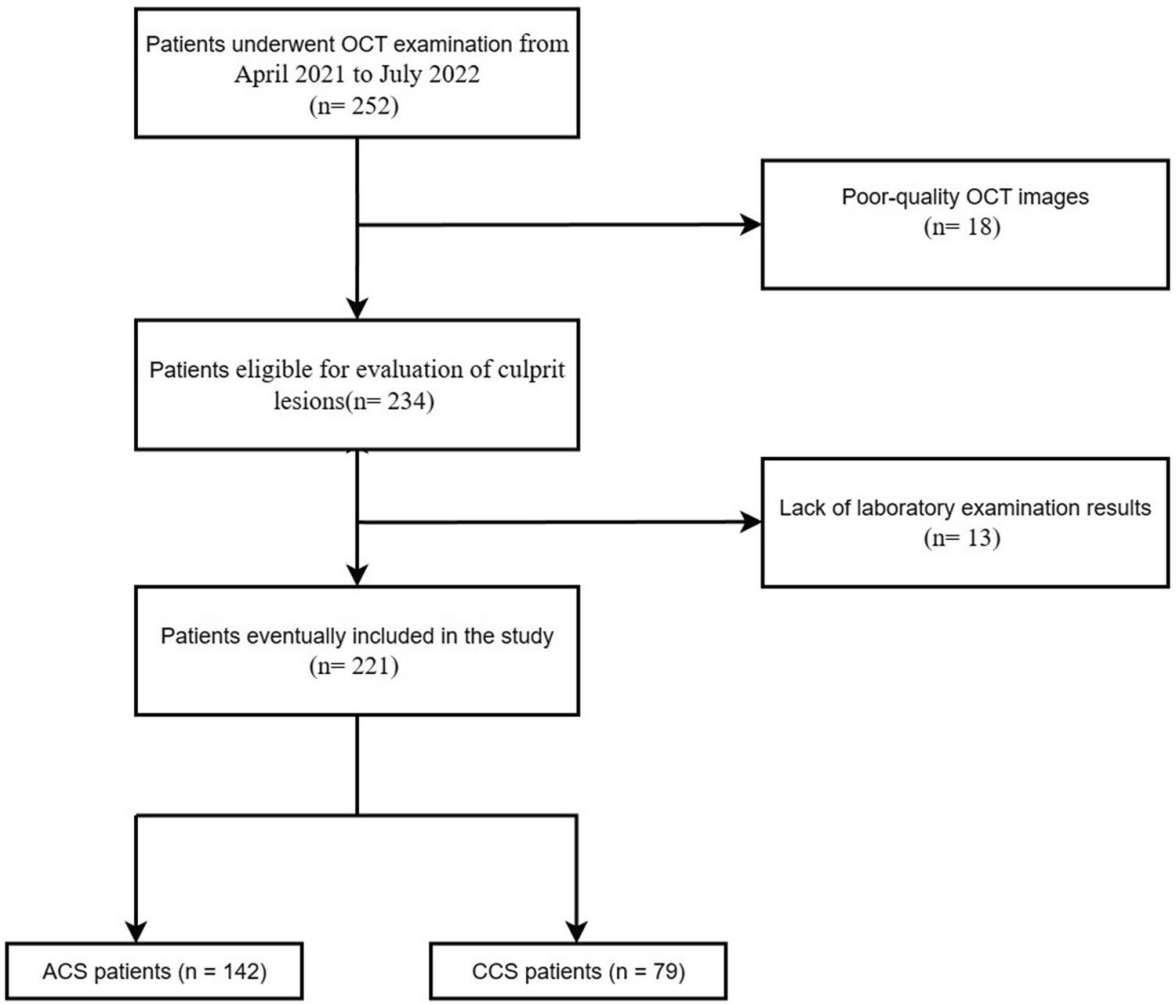
Study flow chart of this study. OCT: optical coherence tomography; ACS: acute coronary syndrome; CCS: chronic coronary syndrome.

The study flowchart is shown in Fig. 1. A total of 252 CAD individuals receiving CAG and OCT examinations were recruited. However, after screening 252 patients, complement C1q values were not obtainable for 13 of these patients, and 18 people were excluded due to the poor quality of OCT examination images. Finally, 221 eligible subjects were included in this research and divided into the ACS (n = 142) and CCS (n = 79) groups based on clinical symptoms, signs, and laboratory testing. The number of patients in unstable angina, STEMI, and NSTEMI groups were 78(54.93%), 29(23.39%), and 35(28.23%) respectively.

Among the 221 eligible patients, 77.83% were male, and the average age was 59.88 ± 10.70 years. Comparing the CCS and ACS groups, the proportion of male patients was predominant, and the average ages were 60.91 ± 9.33 years and 59.25 ± 11.43 years, respectively. The patients' characteristics are shown in Table 1. In the ACS group, the proportion of diabetes mellitus was higher, and the levels of plasma complement C1q of patients in the ACS were also lower, while the levels of total triglycerides (TG), low-density lipoprotein cholesterol (LDL-C), very low-density lipoprotein cholesterol (VLDL-C) and apolipoprotein B (Apo B) were higher. In accordance with expectations, white blood cells, NT-proBNP, and myocardial enzyme levels were considerably higher in the ACS group (*P* < 0.05). In terms of other laboratory variables and angiographic data, including the number of involved vessels, the distribution of culprit vessels, the number of involved vessels, and stent implantation, there were no obvious differences between the two groups (*P* > 0.05), as shown in Table 1 (*P* > 0.05).

**Table 1:** Baseline clinical and angiographic characteristics of the study population.

Characteristics	All (n = 221)	CCS (n = 79)	ACS (n = 142)	*P* value
Age, years, mean ± SD	59.88 ± 10.70	60.91 ± 9.33	59.25 ± 11.43	0.157
Male, n (%)	172 (77.83%)	60 (75.95%)	112 (78.87%)	0.307
Medical history, n (%)				
Hypertension	132 (59.73%)	48 (60.76%)	84 (59.15%)	0.434
Diabetes mellitus	70 (31.67%)	33 (41.77%)	37 (26.06%)	0.017
Smoking	95 (42.99%)	30 (37.97%)	65 (45.77%)	0.151
Drinking	42 (19.00%)	15 (18.99%)	27 (19.01%)	0.733
BMI, kg/m^2^	24.95 ± 3.16	24.66 ± 2.94	25.10 ± 3.27	0.310
Laboratory examination				
WBC, × 109/L	6.91 ± 2.48	6.41 ± 2.22	7.18 ± 2.57	0.014
NEUT, × 109/L	6.14 ± 18.84	4.58 ± 5.26	6.97 ± 22.96	0.006
MO, × 109/L	0.65 ± 1.00	0.57 ± 0.89	0.69 ± 1.06	0.020
LYM, %	26.00 ± 8.65	27.94 ± 8.51	24.97 ± 8.57	0.010
HBA1, %	6.31 ± 1.32	6.51 ± 1.33	6.21 ± 1.31	0.052
TC, mmol/L	3.60 ± 0.99	3.46 ± 0.95	3.67 ± 1.01	0.059
TG, mmol/L	1.68 ± 1.15	1.49 ± 0.81	1.79 ± 1.29	0.050
HDL-C, mmol/L	1.09 ± 0.69	1.10 ± 0.30	1.09 ± 0.83	0.892
LDL-C, mmol/L	2.03 ± 0.88	1.95 ± 0.80	2.47 ± 0.91	0.016
VLDL-C, mmol/L	0.51 ± 0.37	0.43 ± 0.26	0.55 ± 0.41	0.012
Lipoprotein (a)	18.89 ± 25.54	15.67 ± 21.70	20.60 ± 27.27	0.148
Apo A1	1.76 ± 8.67	1.26 ± 0.27	2.03 ± 10.72	0.130
Apo B	0.79 ± 0.27	0.75 ± 0.26	0.82 ± 0.27	0.043
NT-proBNP, pg/ml	2058.13 ± 22,378.24	329.98 ± 1113.44	2970.50 ± 27,636.08	0.002
Hs-cTnI, pg/ml	264.77 ± 929.65	36.81 ± 104.15	382.34 ± 1125.32	< 0.001
LDH, U/L	225.30 ± 147.35	178.95 ± 38.56	249.79 ± 175.29	< 0.001
CK, IU/L	235.90 ± 557.42	119.55 ± 142.08	296.98 ± 673.37	0.018
CK-MB, U/L	28.44 ± 57.23	16.56 ± 13.63	34.67 ± 69.24	0.018
HBDH, IU/L	183.33 ± 162.83	139.68 ± 35.58	208.76 ± 198.93	0.002
AST, IU/L	36.56 ± 54.33	25.13 ± 14.08	42.60 ± 65.66	0.017
TP, g/L	65.37 ± 8.73	66.56 ± 9.90	64.73 ± 8.00	0.022
ALB, g/L	41.46 ± 4.18	42.06 ± 4.48	41.14 ± 3.99	0.039
GLB, g/L	24.32 ± 4.35	25.03 ± 5.13	23.94 ± 3.84	0.059
AG	1.82 ± 1.25	1.73 ± 0.36	1.88 ± 1.52	0.377
UREF, mmol/L	5.42 ± 3.05	4.98 ± 1.45	5.65 ± 3.60	0.103
Creatine, μmol/L	76.49 ± 44.62	68.65 ± 17.15	80.61 ± 53.28	0.010
eGFR, ml/min/1.73m2	105.58 ± 27.30	110.71 ± 26.42	102.84 ± 27.51	0.103
CysC, mg/L	1.52 ± 7.53	0.98 ± 0.19	1.80 ± 9.30	0.418
Uric acid, μmol/L	323.55 ± 94.91	298.20 ± 80.07	337.99 ± 99.81	0.002
C1q, mg/L	18.91 ± 4.01	19.18 ± 4.25	18.25 ± 3.88	0.045
TSH, μIU/ml	3.69 ± 7.06	2.86 ± 1.98	4.10 ± 8.47	0.208
INR	0.96 ± 0.17	0.95 ± 0.19	0.96 ± 0.16	0.666
FIB, mg/dl	169.48 ± 162.75	164.95 ± 157.85	171.84 ± 165.68	0.550
D-dimer, μg/ml	479.35 ± 834.53	425.29 ± 628.36	508.06 ± 926.15	0.461
LVEF, %	62.65 ± 7.97	63.33 ± 7.40	62.30 ± 8.24	0.382
Culprit vessels, n (%)				0.277
LAD	145 (65.61%)	48 (60.76%)	97 (68.31%)	
LCX	27 (12.22%)	14 (17.72%)	13 (9.15%)	
RCA	40 (18.10%)	14 (17.72%)	26 (18.31%)	
Lesion site, n (%)				0.733
Proximal	84 (38.00%)	32 (40.51%)	52 (36.62%)	
Middle	75 (33.94%)	26 (32.91%)	49 (34.51%)	
Distal	53 (23.98%)	18 (22.78%)	35 (24.65%)	
Gensini score	38.27 ± 33.03	34.86 ± 30.77	40.04 ± 34.09	0.241
Stents, n (%)				0.174
0	67 (30.32%)	25 (31.65%)	42 (29.58%)	
1	112 (50.68%)	37 (46.84%)	75(52.82%)	
2	32 (14.48%)	15 (18.99%)	17 (11.97%)	
3	4 (1.81%)	0 (0.00%)	4 (2.82%)	
Medicine				
Statins	205 (92.76%)	65 (82.28%)	140 (98.59%)	0.453
Aspirin	216 (97.74%)	77 (97.47%)	139 (97.89%)	0.512
Anticoagulant	3 (1.36%)	1 (1.27%)	2 (1.41%)	0.925

ACS: acute coronary syndrome, CCS: chronic coronary syndrome, CAD: coronary artery disease, SD: standard deviation, BMI: Body Mass Index, WBC: white blood cell, NEUT: neutrophil, MO: monocyte, HbA1c: glycosylated haemoglobin, TC: total cholesterol, TG: total triglycerides, HDL-C: high-density lipoprotein cholesterol, LDL-C: low-density lipoprotein cholesterol, VLDL-C: very low-density lipoprotein cholesterol, Apo: apolipoprotein, NT-proBNP: N-terminal B-type natriuretic peptide, Hs-cTnI: high-sensitivity cardiac troponin I, CK-MB: creatine kinase, eGFR: estimated glomerular filtration rate, ALT: alanine aminotransferase, AST: aspartate aminotransferase, Cr: creatinine, TSH: thyroid stimulating hormone, LVEF: left ventricular ejection fraction, LAD: left anterior descending artery, LCX: left circumflex artery, RCA: right coronary artery.

### OCT findings of culprit plaques

**Figure 2:**
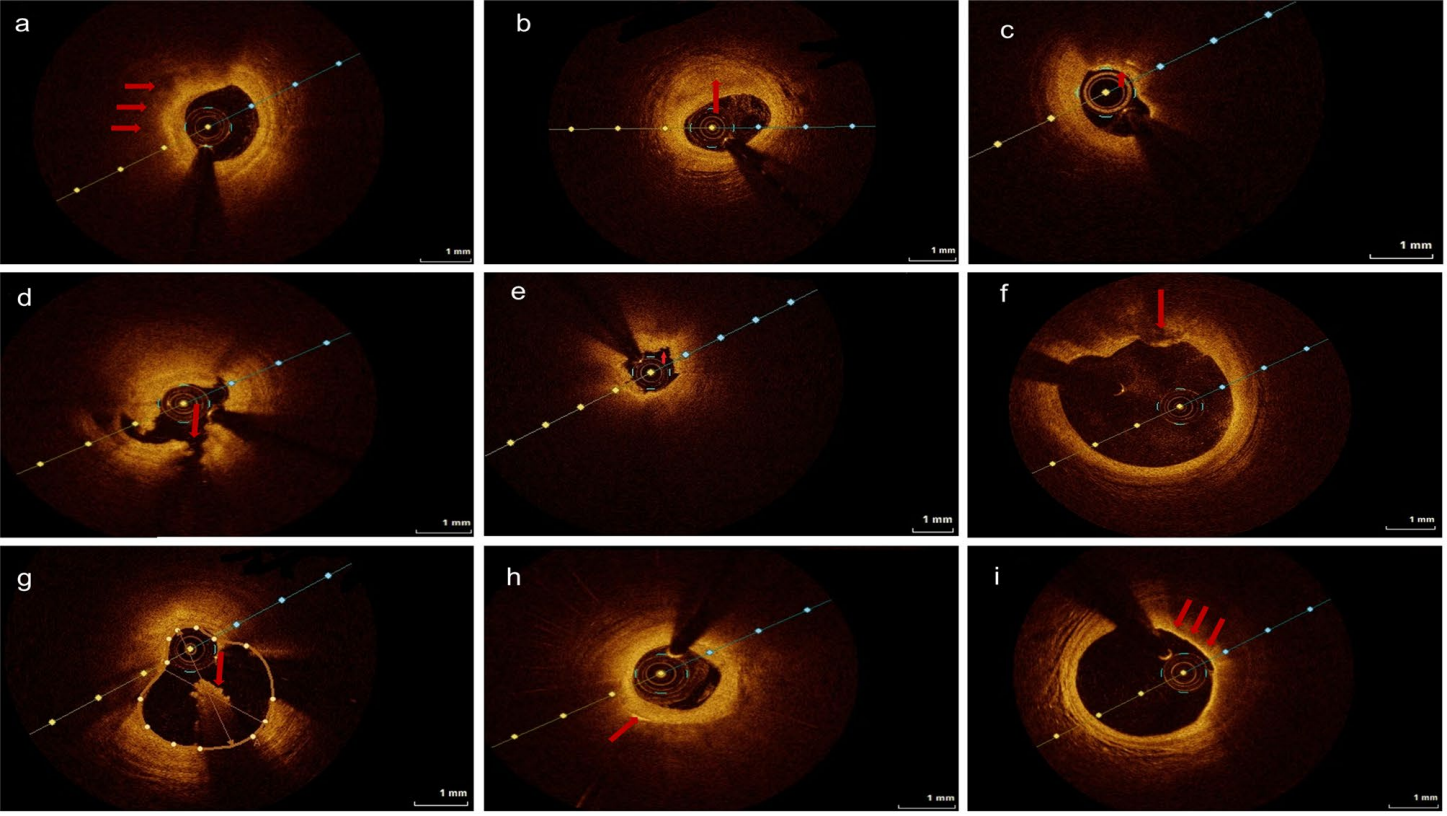
Optical coherence tomography images of a representative cross-section of the culprit lesion. (**a**) Lipid plaques are defined as blurred edges, low signal areas with strong attenuation, and fibrous caps with high signal inside the low-signal areas (arrow). (**b**) Fibrous plaques are defined as uniform, low-attenuation high-signal areas (arrow). (**c**) Thin-cap fibroatheroma (TCFA) is identified as fibrous cap thickness ≤ 65 µm, lipid core larger than two quadrants of lipids, and inflammatory cell infiltration around the fibrous cap (arrow). (**d**) Plaque rupture is defined as a break in the continuity of the fibrous cap of a lipid plaque with cavity formation (arrow). (**e**) Plaque erosion is defined as an intact fibrous cap without plaque rupture with thrombosis and identifiable subthrombotic plaque (arrow). (**f**) Calcified nodules are defined as calcifications that protrude into the lumen of blood vessels with thrombosis (arrow). (**g**) Thrombus are defined as irregularly shaped masses attached to the surface of the coronary lumen or floating in the lumen, including white thrombi, red thrombi, and mixed thrombi. (**h**) Cholesterol crystals are defined as a thin linear region of high signal and low attenuation located at the junction of the fibrous cap and the lipid core (arrow). (**i**) Macrophages are identified as striated structures with high reflected signal and strong attenuation on the fibrous cap and radiolucent shadows below the high signal areas (arrow).

The morphology of the plaques according to OCT data were presented in Fig. 2. A comparison of the OCT features between the two groups is illustrated in Table 2. Notably, the incidence of plaque rupture (27.85 vs. 55.63%, *P* < 0.05), plaque erosion (13.92 vs. 42.25%, *P* = 0.001), and thrombus (20.25 vs. 45.07%, *P* < 0.05) in the culprit lesions of patients with ACS was remarkably higher. In contrast, the fibrous cap thickness of lipid plaques was thicker in patients with CCS (254.31 ± 321.75 vs. 165.38 ± 137.22 µm, *P* < 0.05). Moreover, the incidence of TCFA was generally lower in CCS than in ACS (10.13% vs. 11.27%, *P* = 0.290). In addition, patients with ACS exhibited a higher proportion of other features of vulnerable plaques, such as cholesterol crystals, microvessels, and macrophages, although statistically notable differences were not found (*P* > 0.05). In other types of plaque, such as fibrous plaque and calcified plaque, there was no discernible difference between them. Moreover, compared with the stable plaque groups, the complement C1q levels of the plaque rupture, erosion, thrombus, and cholesterol crystal groups were lower (*P* < 0.05; Table 3).

**Table 2:** Optical coherence tomography characteristics of patients.

Characteristics	All (n = 221)	CCS (n = 79)	ACS (n = 142)	*P* value
Plaque morphology, n (%)				
Plaque rupture	92 (41.62%)	13 (16.46%)	79 (55.63%)	0.008
Plaque erosion	71 (32.13%)	11 (13.92%)	60 (42.25%)	0.001
Calcified nodule	11 (4.98%)	2 (2.53%)	9 (6.34%)	0.098
Plaque type, n (%)				
Thrombus	80 (36.20%)	13 (16.46%)	67 (47.18%)	0.002
Red thrombus	12 (5.43%)	3 (3.80%)	9 (6.34%)	
White thrombus	48 (21.72%)	13 (16.46%)	35 (24.65%)	
Mixed thrombus	20 (9.05%)	4 (5.06%)	16 (11.27%)	
TCFA	24 (10.86%)	8 (10.13%)	16 (11.27%)	0.290
Fibrous plaque	203 (91.86%)	72 (91.14%)	131 (92.25%)	0.663
FCT of fibrous plaque, μm	680.92 ± 761.70	810.35 ± 1238.04	613.05 ± 271.17	0.052
Calcification	89 (40.27%)	35 (44.30%)	54 (38.03%)	0.367
Angle, °	164.70 ± 101.96	155.58 ± 95.08	169.66 ± 105.87	0.502
Thickness, mm	0.99 ± 0.87	1.06 ± 1.39	0.95 ± 0.36	0.520
Length, mm	10.79 ± 19.10	11.92 ± 29.16	10.18 ± 10.41	0.657
Lipid-rich plaque	103 (46.61%)	35 (44.30%)	68 (47.89%)	0.075
FCT, μm	195.98 ± 222.39	254.31 ± 321.75	165.38 ± 137.22	0.003
Lipid arc, °	259.28 ± 82.82	245.79 ± 83.80	267.19 ± 81.50	0.061
Lipid core length, mm	12.34 ± 6.20	11.29 ± 5.84	12.95 ± 6.34	0.055
Lipid index	3501.34 ± 3663.03	3013.09 ± 2308.86	3787.31 ± 4243.09	0.129
Cholesterol crystal	52 (23.53%)	23 (29.11%)	29 (20.42%)	0.146
Microvessel	67 (30.32%)	24 (30.38%)	43 (30.28%)	0.485
Macrophage	116 (52.49%)	43 (54.43%)	73 (51.41%)	0.604
Quantitative of target vessel				
MLA, mm^2^	1.99 ± 1.28	1.99 ± 0.98	1.99 ± 1.41	0.404
MLD, mm	1.35 ± 0.41	1.38 ± 0.36	1.33 ± 0.44	0.123
Diameter stenosis, %	53.31 ± 12.14	53.18 ± 10.95	53.38 ± 12.76	0.889
Area stenosis, %	70.70 ± 14.45	71.68 ± 12.75	70.18 ± 15.28	0.444
Reference vessel diameter, mm	2.92 ± 0.58	2.97 ± 0.58	2.90 ± 0.57	0.356
Reference vessel area, mm^2^	7.12 ± 2.13	7.32 ± 2.88	7.02 ± 2.65	0.668
Post-stent MLA, mm^2^	7.12 ± 2.73	7.32 ± 2.88	7.02 ± 2.65	0.412

*TCFA* thin-cap fibroatheroma.

**Table 3:** Complement C1q level and OCT vulnerable plaque characteristics in patients.

Characteristics	C1q		*P* value
Plaque rupture			
Yes	92	18.72 ± 4.10	
No	129	19.88 ± 3.73	0.008
Plaque erosion			
Yes	71	18.23 ± 3.94	0.019
No	149	19.66 ± 3.99	
Calcified nodule			
Yes	11	20.27 ± 4.79	0.223
No	210	18.77 ± 3.92	
TCFA			
Yes	24	18.11 ± 3.64	0.716
No	196	18.81 ± 4.02	
Fibrous plaque			
Yes	203	18.87 ± 3.93	0.524
No	18	19.48 ± 4.88	
Lipid-rich plaque			
Yes	103	17.95 ± 3.98	0.153
No	116	18.84 ± 3.82	
Calcification			
Yes	89	18.06 ± 3.96	0.310
No	127	17.69 ± 3.99	
Cholesterol crystal			
Yes	52	18.56 ± 3.96	0.021
No	167	19.76 ± 3.91	
Microvessel			
Yes	67	18.81 ± 3.76	0.627
No	153	18.86 ± 4.08	
Thrombus			
Yes	80	18.13 ± 3.74	0.016
No	136	19.87 ± 4.09	
Macrophage			
Yes	116	18.03 ± 3.60	0.476
No	103	18.64 ± 4.38	

### Link between complement C1q and plaque characteristics

Subsequently, we separated the study participants into two groups by the median value of complement C1q levels: low complement-C1q-level group (< 19.10 mg/L, n = 110, 50.0%) and high complement-C1q-level group (≥ 19.10 mg/L, n = 111, 50.0%). The comparison of baseline parameters, angiography, and OCT results is displayed in Table 4. Patients with low complement levels were more prone to develop vulnerable features, including plaque rupture, plaque erosion, thrombus, and cholesterol crystals. No differences were observed in other OCT results or drugs taken (*P* > 0.05).

**Table 4:** Baseline characteristics of patients in the low and high complement C1q groups.

Characteristics	Low C1q (n = 110)	High C1q (n = 111)	*P* value
Age, years	60.22 ± 10.70	59.47 ± 10.81	0.032
Male, n (%)	94 (86.36%)	78 (70.27%)	0.007
Medical history, n (%)			
Hypertension	67 (60.91%)	65 (58.56%)	0.660
Diabetes mellitus	33 (30.00%)	37 (33.33%)	0.626
Family History of CAD	14 (12.73%)	11 (9.91%)	0.493
Smoking	47 (42.73%)	48 (43.24%)	0.985
Drinking	23 (20.91%)	19 (17.12%)	0.452
BMI, kg/m^2^	25.06 ± 3.24	24.86 ± 3.20	0.541
Laboratory examination			
WBC, × 109/L	6.51 ± 2.02	5.02 ± 1.03	0.037
NEUT, × 109/L	7.41 ± 27.91	5.19 ± 4.95	0.084
MO, × 109/L	0.64 ± 1.05	0.53 ± 0.24	0.269
HBA1, %	6.18 ± 0.94	6.38 ± 1.54	0.259
TC, mmol/L	3.74 ± 1.12	3.41 ± 0.77	0.011
TG, mmol/L	1.80 ± 1.14	1.56 ± 0.78	0.071
HDL-C, mmol/L	1.25 ± 0.30	1.04 ± 0.26	0.806
LDL-C, mmol/	2.14 ± 1.01	1.90 ± 0.73	0.039
VLDL-C, mmol/L	0.55 ± 0.36	0.46 ± 0.25	0.028
Lipoprotein (a)	20.53 ± 28.32	16.70 ± 20.70	0.248
Apo A1	1.26 ± 0.27	2.40 ± 13.05	0.012
Apo B	0.83 ± 0.31	0.74 ± 0.21	0.013
NT-proBNP, pg/ml	4800.25 ± 1665.31	3934.88 ± 33,858.11	0.286
Hs-cTnI, pg/ml	266.73 ± 938.90	226.09 ± 1025.19	0.184
LDH, U/L	240.89 ± 167.41	207.22 ± 136.24	0.004
CK, IU/L	262.72 ± 686.67	224.56 ± 454.61	0.087
CK-MB, U/L	32.90 ± 75.27	24.76 ± 36.78	0.309
HBDH, IU/L	197.10 ± 186.47	169.43 ± 136.40	0.038
AST, IU/L	42.00 ± 68.04	32.31 ± 41.26	0.087
TP, g/L	67.16 ± 9.27	63.59 ± 8.31	< 0.001
ALB, g/L	42.30 ± 4.38	40.92 ± 3.85	0.005
GLB, g/L	25.40 ± 4.41	23.03 ± 4.05	< 0.001
AG	1.99 ± 1.83	1.71 ± 0.31	0.022
UREF, mmol/L	5.62 ± 3.92	5.15 ± 2.21	0.279
Cr, μmol/L	82.39 ± 63.60	70.83 ± 18.57	0.012
eGFR, ml/min/1.73m^2^	97.96 ± 28.80	110.26 ± 24.53	0.010
CysC, mg/L	2.18 ± 11.35	0.97 ± 0.24	0.048
Uric acid, μmol/L	336.19 ± 93.90	309.24 ± 94.89	0.034
TSH, μIU/ml	3.12 ± 2.33	3.79 ± 7.65	0.395
INR	0.97 ± 0.22	0.95 ± 0.11	0.329
D-dimer, μg/ml	429.91 ± 663.57	429.82 ± 536.86	0.205
LVEF, %	62.40 ± 7.36	63.43 ± 7.75	0.138
Gensini score	33.17 ± 32.20	35.58 ± 25.90	0.512
Stents, n (%)			
0	39 (35.45%)	28 (25.23%)	
1	56 (50.91%)	56 (50.45%)	
2	13 (17.27%)	13 (11.71%)	
3	4 (3.64%)	0 (0.00%)	
Medicine			
Statins	108 (98.18%)	97 (87.39%)	0.312
Aspirin	107 (97.27%)	109 (98.20%)	0.985
Anticoagulant	2 (1.82%)	1 (0.90%)	0.550
Plaque morphology, n (%)			
Plaque rupture	63 (57.27%)	29 (26.13%)	0.007
Plaque erosion	42 (38.18%)	29 (35.51%)	0.047
Calcified nodule	5 (4.55%)	6 (5.61%)	0.745
Plaque type, n (%)			
Thrombus	47 (42.73%)	33 (29.73%)	0.015
Red thrombus	8 (7.27%)	4 (21.50%)	0.771
White thrombus	27 (24.55%)	21 (32.71%)	0.212
Mixed thrombus	12 (14.81%)	8 (13.08%)	0.714
TCFA	14 (12.73%)	10 (9.01%)	0.373
Fibrous plaque	102 (92.73%)	101 (90.99%)	0.987
FCT of fibrous plaque, μm	647.09 ± 271.31	742.70 ± 1099.04	0.377
Calcification	41 (37.27%)	48 (43.24%)	0.305
Angle, °	165.51 ± 97.37	164.77 ± 104.70	0.973
Thickness, mm	1.02 ± 0.36	1.01 ± 1.23	0.011
Length, mm	13.42 ± 27.94	9.26 ± 11.26	0.251
Lipid-rich plaque	55 (50.00%)	48 (43,24%)	0.165
FCT, μm	173.83 ± 171.60	222.55 ± 272.98	0.034
Lipid arc, °	269.59 ± 80.27	256.06 ± 84.32	0.407
Lipid core length, mm	11.98 ± 6.38	12.58 ± 5.96	0.613
Lipid index	3291.21 ± 2125.01	3155.98 ± 1940.05	0.520
Cholesterol crystal	36 (32.73%)	16 (14.41%)	0.010
Microvessel	36 (32.73%)	31 (27.93%)	0.490
Macrophage	57 (47.27%)	59 (53.15%)	0.728
Quantitative of target vessel			
MLA, mm^2^	2.03 ± 1.11	2.06 ± 1.53	0.713
MLD, mm	1.37 ± 0.40	1.36 ± 0.46	0.489
Diameter stenosis, %	53.35 ± 12.22	53.11 ± 12.31	0.886
Area stenosis, %	71.23 ± 13.83	70.16 ± 13.93	0.469
Reference vessel diameter, mm	2.98 ± 0.61	2.90 ± 0.53	0.304
Reference vessel area, mm2	7.45 ± 2.85	7.00 ± 2.64	0.186

ACS: acute coronary syndrome, CCS: chronic coronary syndrome, CAD: coronary artery disease, SD: standard deviation, BMI: Body Mass Index, WBC: white blood cell, NEUT: neutrophil, MO: monocyte, HbA1c: glycosylated haemoglobin, TC: total cholesterol, TG: total triglycerides, HDL-C: high-density lipoprotein cholesterol, LDL-C: low-density lipoprotein cholesterol, VLDL-C: very low-density lipoprotein cholesterol, Apo: apolipoprotein, NT-proBNP: N-terminal B-type natriuretic peptide, Hs-cTnI: high-sensitivity cardiac troponin I, CK-MB: creatine kinase, eGFR: estimated glomerular filtration rate, ALT: alanine aminotransferase, AST: aspartate aminotransferase, Cr: creatinine, TSH: thyroid stimulating hormone, LVEF: left ventricular ejection fraction, LAD: left anterior descending artery, LCX: left circumflex artery, RCA: right coronary artery, TCFA: thin-cap fibroatheroma.

**Figure 3:**
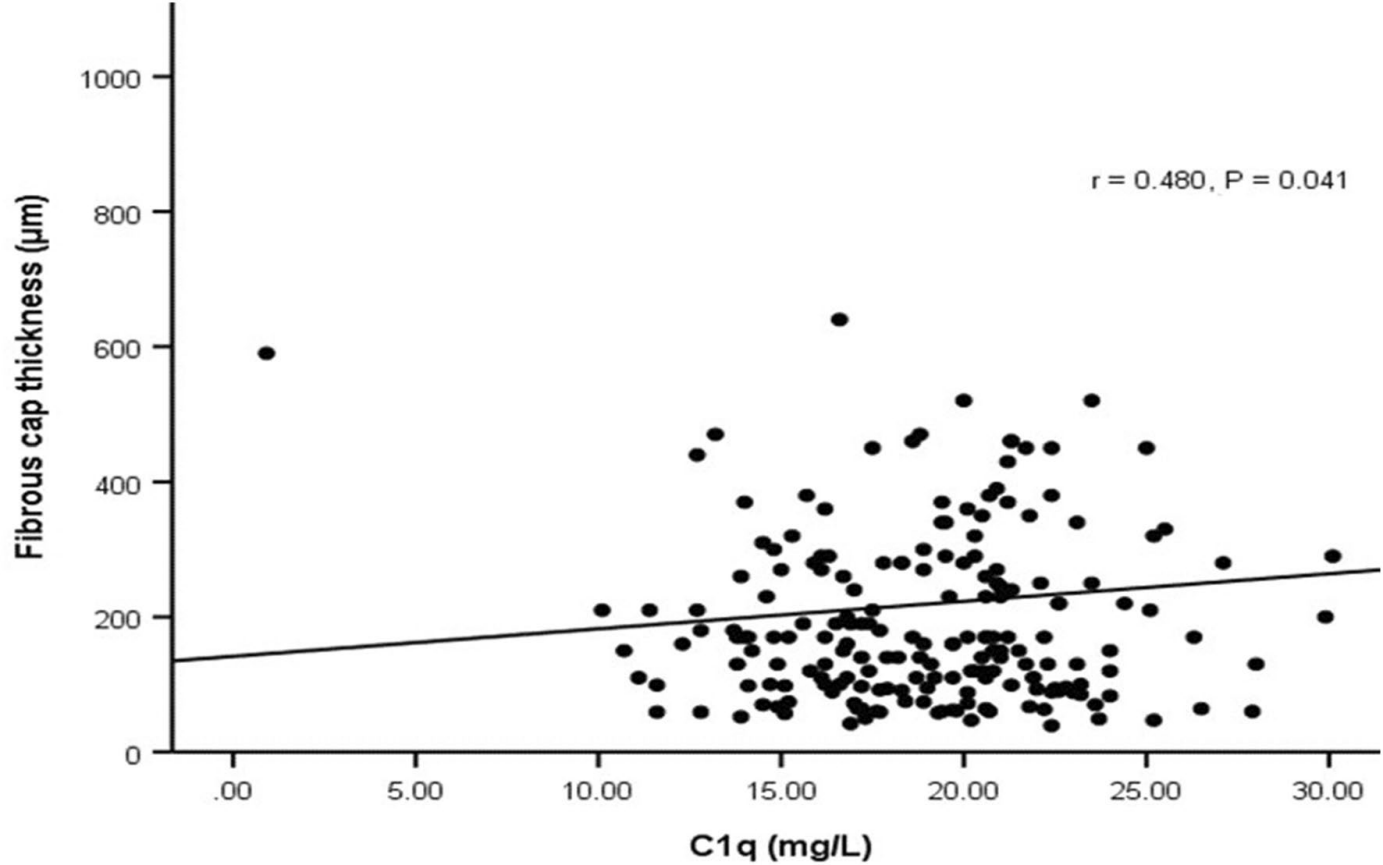
Correlation between complement c1q and fibrous cap thickness in lipid-rich plaque.

We explored the relationship between plasma complement C1q and the FCT of lipid-rich plaques. Spearman analysis indicated a positive correlation between them (r = 0.480, *P* = 0.041) (Fig. 3). In lipid-rich plaques, the fibrous cap thickness increases with the increase of complement C1q level, indicating that complement C1q could improve the stability of lipid-rich plaques. Univariate logistic regression analysis indicated that the complement C1q level was related to plaque rupture (Table 5), erosion (Supplementary Table S1), thrombus (Supplementary Table S2), and cholesterol crystals (Supplementary Table S3). After adjusting for confounding factors, such as age, sex, alcohol drinking, smoking, TC, LDL-C and Apo B in different models for multivariate logistic regression analysis, the complement C1q remained detective for plaque rupture, erosion, thrombus and cholesterol crystals (*P* < 0.05).

**Table 5:** Logistic regression analysis of plaque rupture.

Variables	OR	95% CI	*P* value
C1q	0.571	0.121–0.677	0.001
TG	1.161	0.930–1.482	0. 177
TC	1.410	1.130–2.010	0.005
HDL-C	0.438	0.282–1.685	0.481
LDL-C	1.518	1.198–2.350	0.003
VLDL-C	1.336	0.884–2.865	0.358
Lipoprotein (a)	1.002	1.000–1.010	0.072
Apo A1	0.642	0.274–1.532	0.086
Apo B	4.580	1.161–14.180	0.004
Model 1	0.664	0.117–0.883	0.050
Model 2	0.498	0.115–0.686	0.011
Model 3	0.561	0.073–0.718	0.001
Model 4	0.514	0.141–0.687	0.010

Model 1: C1q, Sex, Age.

Model 2: C1q, Age, Sex, TC.

Model 3: C1q, Sex, Age, TC, LDL-C.

Model 4: C1q, Sex, Age, TC, LDL-C, Apo B.

**Figure 4:**
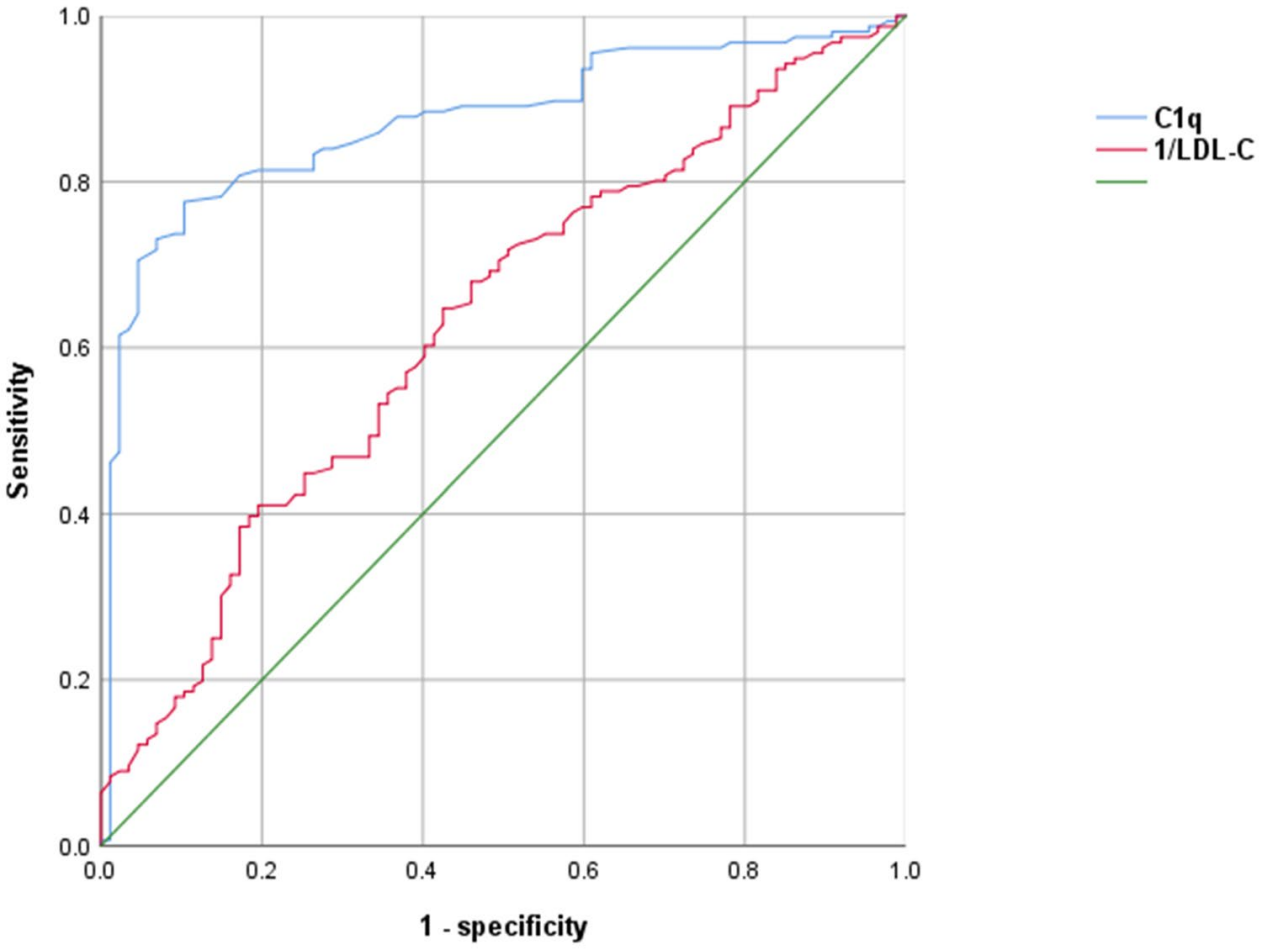
Receiver operating characteristic (ROC) curves that distinguish between the rupture and non-rupture groups. AUC: area under the curve, LDL-C: low-density lipoprotein cholesterol.

The ROC curve was further used to investigate whether complement C1q and LDL-C could be marker for the detection of plaque vulnerability. The area under the ROC curve for complement C1q and 1/LDL-C in the plaque rupture group were 0.873 (95% CI 0.827–0.918, *P* = 0.023) and 0.630 (95% CI 0.557–0.702, *P* = 0.037). Youden's Index analyses yielded the optimal cut-off values for complement C1q and LDL-C were 18.9 mg/L and 2.21 mmol/L, respectively, which corresponded to sensitivities and specificities of 0.78/0.90 for complement C1q and 0.67/ 0.59 for LDL-C. (Fig. 4). In the plaque erosion group, the AUCs were 0.816 (95% CI 0.756–0.876, *P* = 0.001) for complement C1q and 0.704 (95% CI 0.628–0.779, *P* = 0.001) for 1/LDL-C, Youden's Index analyses yielded the optimal cut-off values for complement C1q and LDL-C were 18.75 mg/L and 2.35 mmol/L, respectively, which corresponded to sensitivities and specificities of 0.84/0.75 for complement C1q and 0.74/ 0.67 for LDL-C. (Supplementary Fig. S1). In the thrombus group, the AUC was 0.785 (95% CI 0.722–0.849, *P* = 0.032) for complement C1q and 0.695 (95% CI 0.624–0.767, *P* = 0.037) for 1/LDL-C. Youden's Index analyses yielded the optimal cut-off values for complement C1q and LDL-C were 18.05 mg/L and 2.39 mmol/L, respectively, which corresponded to sensitivities and specificities of 0.80/0.80 for complement C1q and 0.78/ 0.57 for LDL-C. (Supplementary Fig. S2). In the cholesterol crystal group, the AUC was 0.837 (95% CI 0.769–0.905, *P* = 0.035) for complement C1q and 0.747 (95% CI 0.677–0.818, *P* = 0.036) for 1/LDL-C. Youden's Index analyses yielded the optimal cut-off values for complement C1q and LDL-C were 18.65 mg/L and 2.48 mmol/L, respectively, which corresponded to sensitivities and specificities of 0.85/0.80 for complement C1q and 0.68/0.76 for LDL-C. (Supplementary Fig. S3).

## Discussion

CAD is a multifactorial atherosclerotic disease, some factors contributing to it or aggravating the condition including chronic inflammation, mitochondrial dysfunction, endothelial dysfunction, dyslipidaemia, hypertension, metabolic syndrome, obesity, and type 2 diabetes mellitus (T2DM)^8,13–19^. Atherosclerosis is caused by a variety of immune system effects in the circulation and vascular lesion sites^20^, and complement C1q is an important part of the immune system which has a vital role in the progression of atherosclerosis. Nevertheless, few studies are available on complement C1q in CAD patients. Therefore, we evaluated the correlation between plasma C1q levels and plaque vulnerability in patients with CAD in this research.

The inflammatory response has an irreplaceable impact on the formation and progression of atherosclerotic lesions, which ultimately leads to plaque instability which relates to CAD^8^. In advanced stages of atherosclerosis, the formation of the C1 complex (C1qC1r2C1s2) triggers the classical pathway of the proinflammatory complement cascade and leads to disease development^21^, while during the early stage, C1q has a protective role in maintaining normal tissue homeostasis by improving macrophage foam cell survival and function during the removal of modified lipoproteins^22^. The CODAM study found that low C1q levels were related to a higher prevalence of CAD^23^. Cavusoglu found that reductions in C1q levels were associated with increased risk for all-cause mortality at 10 years among diabetes patients with known or suspected coronary artery disease^24^. According to a cross-sectional investigation, patients with severe coronary artery stenosis had considerably lower serum C1q concentrations than those with no stenosis or mild to moderate stenosis^25^. In this retrospective study, one of our findings is that plasma complement C1q may be a prediction of types in patients with CAD. The value of plasma complement C1q was notably lower in patients with ACS than in those with CCS, which supported evidence from previous observational studies^26,27^.

Classical complement pathway activation plays an essential role in modulating the development of atherosclerotic plaques. Complement C1q plays a beneficial role by mediating phagocytosis of cholesterol crystals, facilitating the removal of cholesterol crystals in inflammatory atherosclerotic plaques^28^. Additionally, C1q regulates phagocyte polarization to an anti-inflammatory m2-like phenotype during the clearance of apoptotic cells, reduces lipid accumulation, and promotes plaque stabilization^29^. To further explore the correlation between complement C1q and the vulnerability of coronary plaques in CAD patients, we compared the serum concentration of complement C1q in CAD patients with different plaque types. The results showed that the level of C1q was lower in patients with plaque rupture, erosion, thrombus, and cholesterol crystals except for calcified nodules, revealing that complement C1q may be linked to the stability of coronary plaques. The low concentrations of complement C1q may served as a reliable predictor of vulnerable plaque, consistent with the literature^25,27^.

We also found that the complement C1q has a positively correlation with FCT in lipid-rich plaque, indicating the higher complement C1q value, the more stable status of TCFA in lipid-rich plaques. Elevation of the complement C1q level may decrease the incidence of TCFA and ACS, which requires further work for verification. A logistic regression model was constructed to further investigate the relationship between C1q values and coronary plaque vulnerability in CAD patients and found that C1q values were correlated with a decreased OR of plaque rupture, erosion, thrombus, and cholesterol crystals. In our research, the ROC curves of complement C1q and LDL-C demonstrated good sensitivity and specificity for coronary vulnerable plaques. Furthermore, complement C1q's AUC of the ROC curve is greater than that of 1/LDL-C, the area under the ROC curve for complement C1q and 1/LDL-C were 0.873 vs 0.630, 0.816 vs 0.704, 0.785 vs 0.695, 0.837 vs 0.747 in the plaque rupture, erosion, thrombus and cholesterol crystal group respectively.Through the analysis of the above findings, complement C1q possibly evaluates plaques’ stability better than LDL-C in patients with CAD, which means the level of complement C1q may be an better biomarker of vulnerable plaque compared with LDL-C. Complement C1q may serve as a valuable indicator for detecting ACS events and providing supplementary information to disease assessment rather than be utilized as a standalone diagnostic marker for ACS.

### Study strengths and limitations

Our study is the first investigation to show the association of complement C1q with coronary plaque stability by OCT imaging in patients with coronary heart disease. In this study, we firstly found that there are differences in serum complement levels in different diseased populations, and further analyzed the relationship between complement levels and different plaque types by logistic regression and other methods, which morphologically proved the correlation between complement levels and vulnerable plaques. Furthermore, due to its high cost, invasiveness and complexity, OCT is not suitable for widespread and repeated use in the population. This study provided more evidence for the non-invasive analysis of coronary vulnerable plaques distribution and evaluation of patients' prognosis. However, our findings have limitations. First, this is a retrospective, single-center study, the sample size was limited. Second, patients with low-risk NSTEMI and unstable angina who underwent antithrombotic agent treatments before PCI were enrolled in the analysis. Undergoing those medical therapies before catheterization may have dissolved thrombi and altered the results of OCT. Third, OCT is routinely performed in culprit vessel, and OCT imaging data from other coronary branches are not available. There is no clear clinical evidence that non-culprit lesions affect complement levels in patients. Therefore, we will continue to investigate the possible relationship between complement C1q levels and plaque characteristics in other non-culprit vessels in ACS patients. Finally, since the data are derived from electronic medical records, classification errors and misdiagnoses are inevitable. Despite the cleaning and standardization of our data, some minor confounding factors may still affect the stability of the results. For example, one confounding factor is that risk factors are typically measured just once; therefore, the observed correlation will merely represent a unique timepoint estimate and will thus raise the issue of regression dilution bias.

## Conclusion

In conclusion, we found that the complement C1q was lower in ACS patients than that of CCS patients, and serum complement C1q was linked to various types of culprit plaques in the coronary arteries, including plaque rupture, plaque erosion, thrombus, and cholesterol crystal. In patients with CAD, the complement C1q value could be a reliable indicator of coronary vulnerable plaques.

### Supplementary Information

Supplementary Information 1.

Supplementary Information 2.

Supplementary Information 3.

Supplementary Information 4.

Supplementary Information 5.

## Data Availability

The datasets utilized and/or analyzed during the current study are available from the corresponding author upon reasonable request.

## Abbreviations

CAD: Coronary artery disease
ASCVD: Atherosclerotic cardiovascular disease
ACS: Acute coronary syndrome
CCS: Chronic coronary syndrome
AS: Atherosclerosis
DM: Diabetesmellitus
OCT: Optical coherence tomography
LDL-C: Low-density lipoprotein cholesterol
HDL-C: High-density lipoprotein cholesterol
WBC: White blood cells
TC: Total cholesterol
TG: Total triglycerides
CAG: Coronary angiography
PCI: Percutaneous coronary intervention
hs-cTnI: High-sensitivity cardiac troponin I
FCT: Fibrous cap thickness
TCFA: Thin-cap fbroatheroma
ROC: Receiver operator characteristic
AUC: Area under the curve
NSTEMI: Non-ST-segment elevation myocardial infarction
