# Supplementary figures and images for: The relationship between complement C1q and coronary plaque vulnerability based on optical coherence tomography analysis
Source: Sci Rep. 2024 Apr 25;14:9477. doi: 10.1038/s41598-024-60128-0 (PMC11043360; doi:10.1038/s41598-024-60128-0)

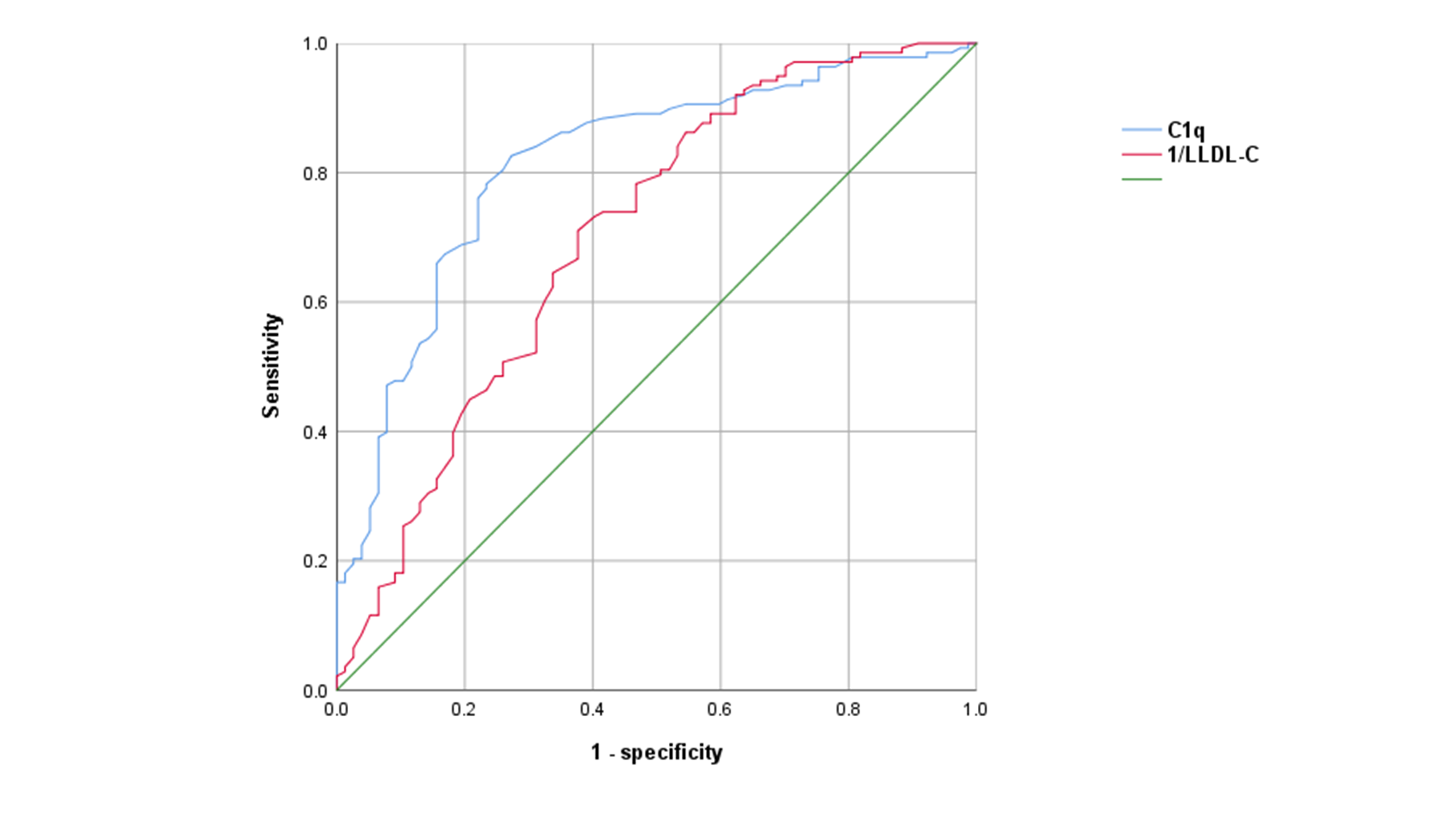

Supplement: Supplementary file 1 — Supplementary Information 1. [file 41598_2024_60128_MOESM1_ESM.zip › articlefigure216_3.tif]

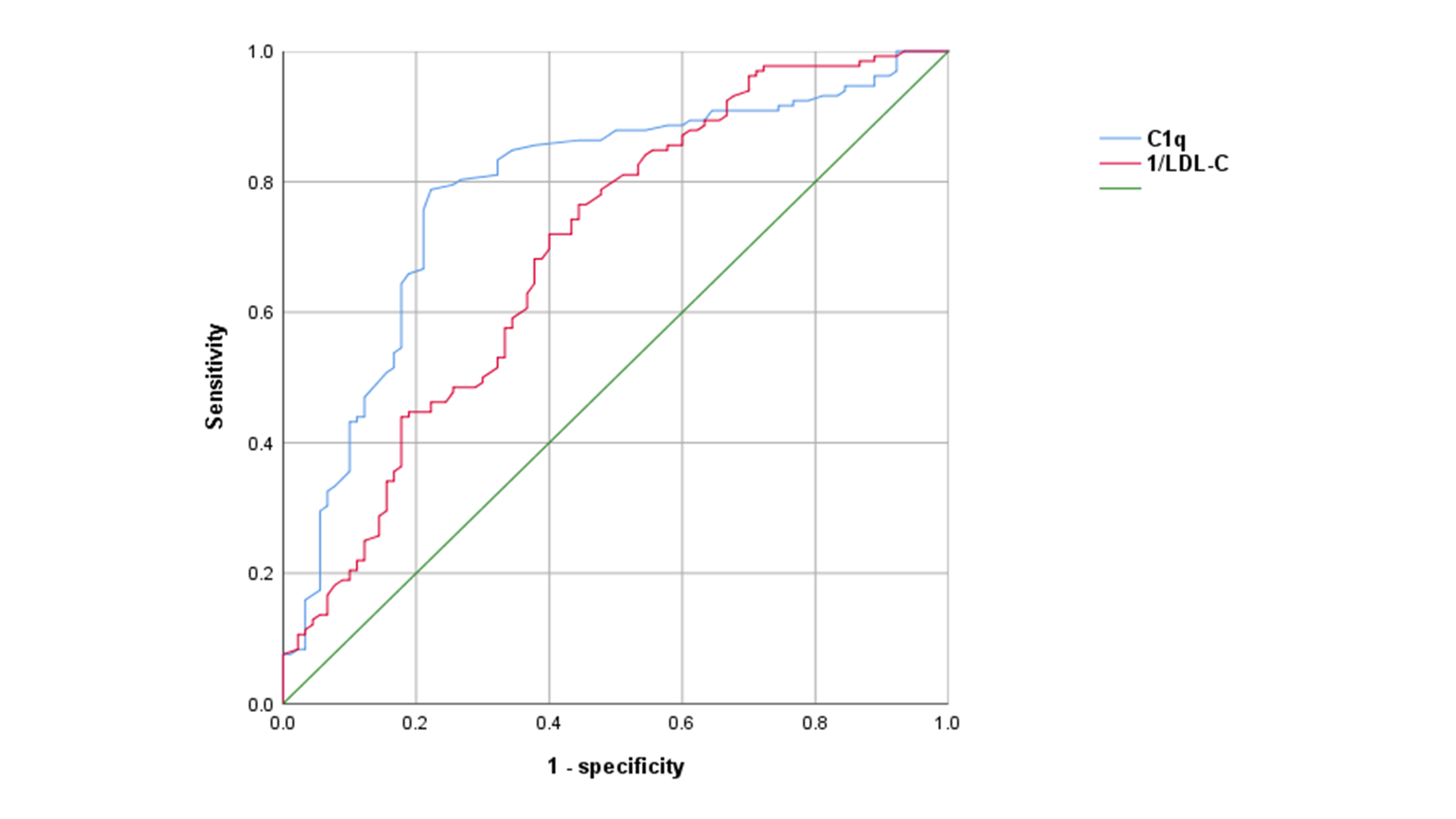

Supplement: Supplementary file 2 — Supplementary Information 2. [file 41598_2024_60128_MOESM2_ESM.zip › articlefigure216_5.tif]

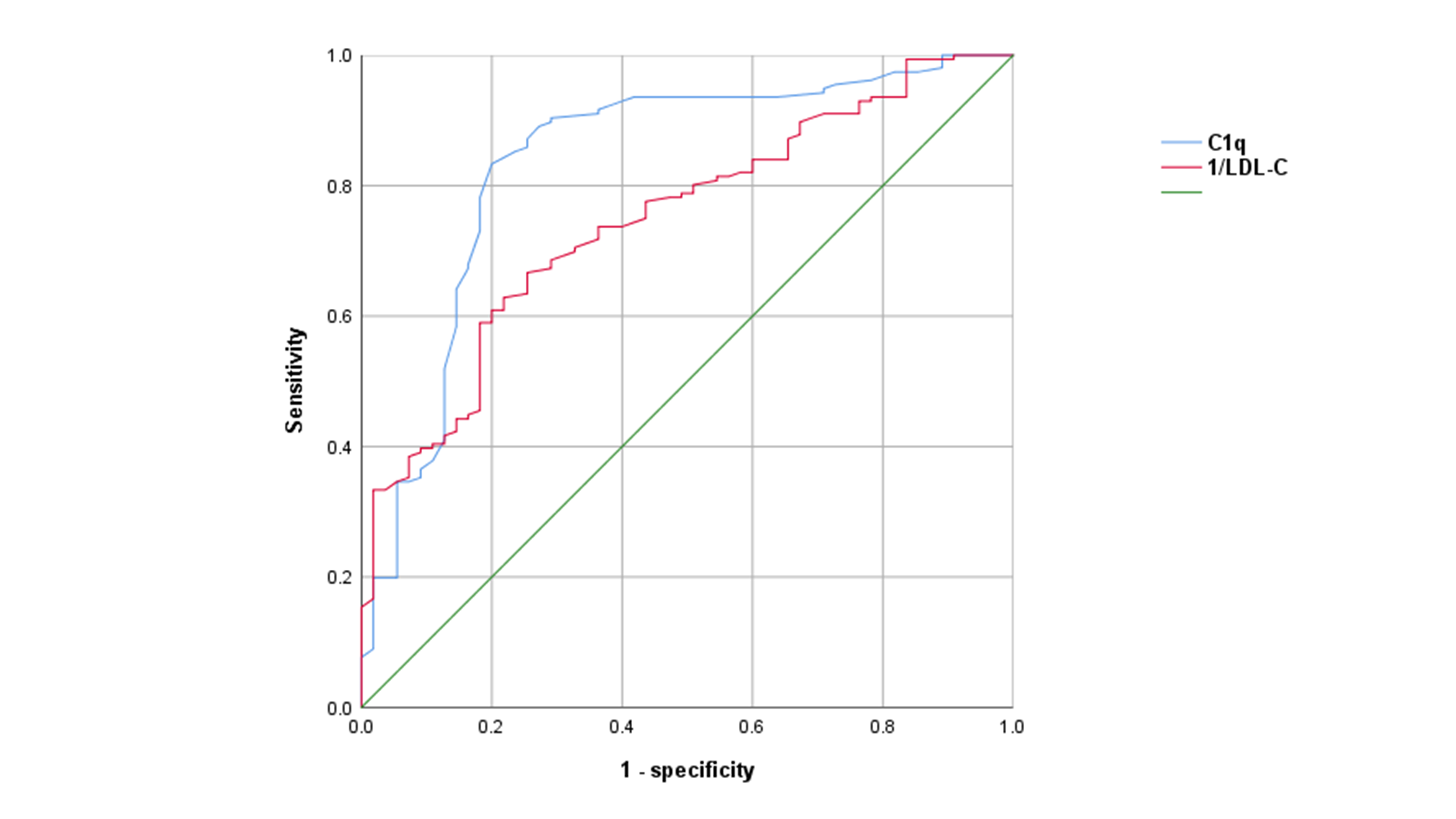

Supplement: Supplementary file 3 — Supplementary Information 3. [file 41598_2024_60128_MOESM3_ESM.zip › articlefigure216_7.tif]
