# Supplementary material for: The relationship between complement C1q and coronary plaque vulnerability based on optical coherence tomography analysis
Source: Sci Rep. 2024 Apr 25;14:9477. doi: 10.1038/s41598-024-60128-0 (PMC11043360; doi:10.1038/s41598-024-60128-0)
Supplement: Supplementary file 4 — Supplementary Information 4. [file 41598_2024_60128_MOESM4_ESM.pdf]

Table S1 Logistic regression analysis of plaque erosion

Variables	OR	95% CI	P value
C1q	0.453	0.171 – 0.787	0.021
TG	1.037	0.825 – 1.305	0.750
TC	1.336	1.052-1.863	0.021
HDL-C	0.648	0.289-1.305	0.452
LDL-C	1.537	1.217-2.406	0.022
VLDL-C	1.090	0.428-1.954	0.817
Lipoprotein (a)	1.003	0.986-1.008	0.618
Apo A1	0.835	0.778-6.829	0.132
Apo B	1.349	1.320-11.243	0.014
Model 1	0.482	0.119-0.854	0.015
Model 2	0.669	0.173-0.962	0.020
Model 3	0.542	0.062-0.893	0.003
Model 4	0.480	0.101-0.731	0.010

Model 1: C1q, Sex, Age

Model 2: C1q, Sex, Age, Hypertension, Smoking

Model 3: C1q, Sex, Age, Hypertension, Smoking, LDL-C

Model 4: C1q, Sex, Age, Hypertension, Smoking, LDL-C, Apo B

Table S2 Logistic regression analysis of thrombus

Variables	OR	95% CI	P value
C1q	0.271	0.014 – 0.677	0.001
TG	1.144	0.917 – 1.456	0.221
TC	1.396	1.118-1.976	0.006
HDL-C	0.729	0.776-1.670	0.508
LDL-C	1.487	1.167-2.268	0.004
VLDL-C	1.395	0.723-3.049	0.281
Lipoprotein (a)	1.001	0.989-1.009	0.824
Apo A1	0.775	0.451-6.278	0.152
Apo B	3.457	1.493-12.357	0.007
Model 1	0.323	0.011-0.826	0.010
Model 2	0.215	0.018-0.929	0.005
Model 3	0.248	0.032-0.793	0.001

Model 1: C1q, Sex, Age

Model 2: C1q, Sex, Age, LDL-C

Model 3: C1q, Sex, Age, LDL-C, Apo B

Table S3 Logistic regression analysis of cholesterol crystal

Variables	OR	95% CI	P value
C1q	0.686	0.171–0.977	0.006
TG	1.091	0.860–1.394	0.460
TC	1.220	0.915-1.696	0.164
HDL-C	0.409	0.124-1.807	0.320
LDL-C	1.583	1.234-2.600	0.002
VLDL-C	1.588	0.197-1.563	0.265
Lipoprotein (a)	1.001	0.989-1.012	0.922
Apo A1	0.418	0.125-1.077	0.071
Apo B	5.312	1.155-11.942	0.028
Model 1	0.709	0.135-0.916	0.005
Model 2	0.624	0.056-0.934	0.010
Model 3	0.564	0.117-0.882	0.025
Model 4	0.472	0.013-0.890	0.001

Model 1: C1q, Age,Sex

Model 2: C1q, Age, Sex,TC

Model 3: C1q, Age, Sex,TC, LDL-C

Model 4: C1q, Age, Sex,TC, LDL-C, Apo B
