# Supplementary material for: The relationship between complement C1q and coronary plaque vulnerability based on optical coherence tomography analysis
Source: Sci Rep. 2024 Apr 25;14:9477. doi: 10.1038/s41598-024-60128-0 (PMC11043360; doi:10.1038/s41598-024-60128-0)
Supplement: Supplementary file 5 — Supplementary Information 5. [file 41598_2024_60128_MOESM5_ESM.pdf]

Supplementary figure legends

Fig. S1 Receiver operating characteristic (ROC) curves that distinguish between the erosion and non-erosion groups.

AUC: area under the curve, LDL-C: low-density lipoprotein cholesterol

Fig. S2 Receiver operating characteristic (ROC) curves that distinguish between the thrombus and non-thrombus groups.

AUC: area under the curve, LDL-C: low-density lipoprotein cholesterol

Fig. S3 Receiver operating characteristic (ROC) curves that distinguish between the cholesterol crystal and non-cholesterol groups.

AUC: area under the curve, LDL-C: low-density lipoprotein cholesterol
